# Hematoma in the Bucco-Mandibular Space: First Case Report

**DOI:** 10.7759/cureus.1771

**Published:** 2017-10-12

**Authors:** Joe Iwanaga, Charlotte Wilson, Emre Yilmaz, Cameron K Schmidt, Rod J Oskouian, R. Shane Tubbs

**Affiliations:** 1 Seattle Science Foundation; 2 Swedish Medical Center, Swedish Neuroscience Institute; 3 Clinical Anatomy, Seattle Science Foundation; 4 Neurosurgery, Complex Spine, Swedish Neuroscience Institute; 5 Neurosurgery, Seattle Science Foundation

**Keywords:** mimetic muscle, mandible, anatomy, cadaver, fascia

## Abstract

Our previous studies based on intraoral dissection of fresh cadavers revealed that the fissure and loose connective tissues deep to the mucosa between the incisivus labii inferioris muscle and buccinator muscle form the entrance of the newly discovered bucco-mandibular space. To support the clinical significance of this space, we report the finding of a hematoma within this space in an adult fresh cadaver. Such a finding lends credence to studying the bucco-mandibular space and might help better understand the spread of some infections in the oral region.

## Introduction

Our previous studies based on intraoral dissection of fresh cadavers revealed that the fissure and loose connective tissues deep to the mucosa between the incisivus labii inferioris (ILI) muscle and buccinator muscle (BM) make entrance of the newly discovered space which is named the “bucco-mandibular space” [1]. This space is bounded by the ILI and mentalis anteriorly, anterior margin of the masseter muscle and its fascia posteriorly, depressor anguli oris (DAO) inferiorly and laterally, lateral surface of the mandible medially, and platysma and associated fascia, which is continuous with the masseteric fascia superiorly.

We present a case of hematoma found in the bucco-mandibular space on the left side of the mandible in a postmortem specimen.

## Case presentation

**Figure 1:**
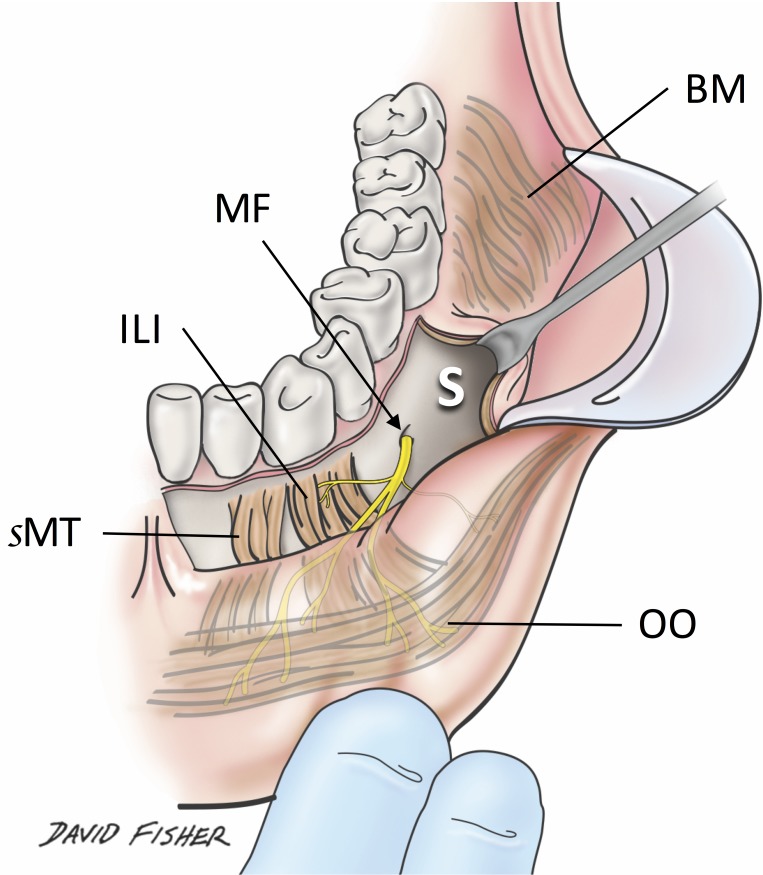
Bucco-mandibular space. BM: buccinator muscle; ILI: incisivus labii inferioris; MF: mental foramen; sMT: superior portion of mentalis; OO: orbicularis oris; S: bucco-mandibular space.

**Figure 2:**
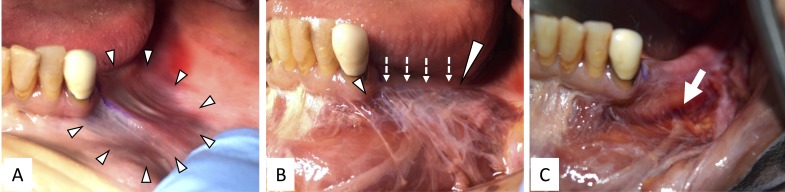
Hematoma in the bucco-mandibular space over left mandible. A: Hematoma in the buccal side of the mandible (arrowheads). B: After removing the mucosa on the hematoma. Note that thin connective tissue (white dotted arrows) existed on the hematoma only between lateral border of the incisivus labii inferioris (short arrowhead) and anterior border of the buccinators (long arrowhead) muscles. C: After removing thin connective tissue on the hematoma. Note that the mental foramen (arrow) and its nerve are located deep to the hematoma.

A hematoma was found on the left side of the mandible in female Caucasian cadaver whose age at death was 82 years. The range of the lesion was canine anteriorly (slightly anterior to the buccal frenulum) and the first to second molar posteriorly. The mucosa was removed in order to observe the relationship between the hematoma and mimetic muscles. The hematoma spread onto the ILI anteriorly, to anterior border of the masseter muscle posteriorly underneath the BM, and to DAO inferolaterally. Thus, this hematoma was restricted in the bucco-mandibular space (Figure 1). The mental foramen and the existing mental nerve were positioned between the ILI and BM (Figure 2).

All dissections were performed under a surgical microscope (OPMI CS NC31, Carl Zeiss, Oberkochen, Germany). The present study protocol did not require approval by the ethics committees of our institutions, and work was performed in accordance with the requirements of the Declaration of Helsinki (64th WMA General Assembly, Fortaleza, Brazil, October 2013).

## Discussion

The case reported herein clearly demonstrated the bucco-mandibular space and related pathology. However, we need to understand all the related mimetic muscles three dimensionally prior to discussing this space, because most of those mimetic muscles related to the oral cavity have been described via dissection from outside the face and not from an intraoral perspective. Also, most of the studies of the mimetic muscles used embalmed cadavers where it is difficult to retract the muscles and other soft tissues [2,3].

The anterior border of the BM corresponds to the buccal frenulum (which means when the buccal membrane is retracted laterally) around the second premolar to the first molar region [4]. Also, the bony attachment of the BM corresponds to the mucogingival junction [5]. According to textbook descriptions [6] and anatomical studies of the ILI [2,3,5], the ILI origins at the same level as the mucogingival junction of the mandible pass from the alveolar border of the mandible between the central incisor medially and canine laterally and toward the angle of the mouth or inferior part of the orbicularis oris muscle obliquely. These two muscles, the BM and ILI, thus form the entrance into the bucco-mandibular space. This space consists of loose connective tissue so that once bleeding or infection accumulates here, it can spread easily. Based on our previous study, this space can harbor 20 to 40 cc of volume [1].

In this case, the hematoma was found incidentally in the postmortem mandible, and mental foramen and related nerve were included deep to this hematoma, which means some pathological changes in this space could compress or damage the foramen and nerve. Since hypesthesia or anesthesia of the lower lip as a sign of inflammation of the ipsilateral mandible was first described by Vincent [7] in 1896, it has been well recognized in osteomyelitis and other infectious conditions of the mandible and often described as “Vincent’s Syndrome” [8].

We could diagnose this symptom as a result of the inflammation when we know the osteomyelitis or other infection occurring in the mandible.

## Conclusions

Based on this report and our previous studies, dentists should be aware that not only the inflammation inside the mandible but the hematoma outside the mandible and other infectious conditions occurring in this space might result in Vincent's syndrome.
